# Model checking in multiple imputation: an overview and case study

**DOI:** 10.1186/s12982-017-0062-6

**Published:** 2017-08-23

**Authors:** Cattram D. Nguyen, John B. Carlin, Katherine J. Lee

**Affiliations:** 1 Clinical Epidemiology and Biostatistics Unit, Murdoch Childrens Research Institute, The Royal Children’s Hospital, Flemington Road, Parkville, VIC 3052 Australia; 2 Department of Paediatrics (RCH Academic Centre), Faculty of Medicine, Dentistry and Health Sciences, The Royal Children’s Hospital, University of Melbourne, Flemington Road, Parkville, VIC 3052 Australia

**Keywords:** Missing data, Model checking, Multiple imputation, Posterior predictive checking, Cross-validation, Diagnostics

## Abstract

**Background:**

Multiple imputation has become very popular as a general-purpose method for handling missing data. The validity of multiple-imputation-based analyses relies on the use of an appropriate model to impute the missing values. Despite the widespread use of multiple imputation, there are few guidelines available for checking imputation models.

**Analysis:**

In this paper, we provide an overview of currently available methods for checking imputation models. These include graphical checks and numerical summaries, as well as simulation-based methods such as posterior predictive checking. These model checking techniques are illustrated using an analysis affected by missing data from the Longitudinal Study of Australian Children.

**Conclusions:**

As multiple imputation becomes further established as a standard approach for handling missing data, it will become increasingly important that researchers employ appropriate model checking approaches to ensure that reliable results are obtained when using this method.

**Electronic supplementary material:**

The online version of this article (doi:10.1186/s12982-017-0062-6) contains supplementary material, which is available to authorized users.

## Background

Missing data are a pervasive problem in medical and epidemiological research. In recent years there have been advances in missing data methods, as well as increased recommendations from scientific journals to apply principled methods to incomplete data problems [1]. One of the commonly used methods for handling missing data is multiple imputation (MI). Under this approach each missing value in the dataset is replaced with an imputed value; this process is repeated with an element of randomness resulting in multiple “completed” datasets, each consisting of observed and imputed values. Standard analysis methods are then applied to each of the completed datasets, and the results are combined using simple formulae (known as Rubin’s rules) to give final estimates of target parameters with standard errors that appropriately allow for the uncertainty of the missing data [2].

MI has become very popular, as it can provide gains over analyses that only include data from participants with completely observed data (known as complete case analyses). MI does not suffer from the same losses of information as complete case analyses, because it can use information from cases with partially observed data, and it also has the potential to correct for bias associated with the omission of incomplete cases [3, 4].

In order for MI to produce valid results, the imputations must be generated using a sensible process. The most challenging task when using MI is the specification of the model for producing the imputed values (generally referred to as the “imputation model”). When constructing imputation models, imputers need to make several decisions concerning, for example, the functional form of the imputation model [5], the selection of variables to include in the model [6], possible methods for accommodating non-linear relationships [7], and how best to impute categorical and non-normal continuous variables [8–10]. In many cases, there is no consensus in the literature to inform these modelling decisions. If the imputation model is poorly specified (such as through the omission of variables that appear in the subsequent analysis model), this can lead to invalid estimates of the target parameters. Given that best practice guidelines for MI are still evolving, it can be challenging for researchers to avoid pitfalls in imputation modelling [3].

The validity of MI also rests on assumptions concerning the missing data mechanisms, i.e. the processes underlying how the missing data arose. Most standard implementations of MI assume that the unobserved values are missing at random (MAR), i.e. that the probability of non-response depends on the observed data and not on the values of the missing data. The assumption of MAR is fundamental to most implementations of MI, as it enables the imputations to be generated without explicitly modelling the missing data process.

As with all statistical models, it is important that researchers perform checks of their imputation models to examine how the results of the desired analysis may be affected by the specified imputation model. Despite the popularity of MI, the checking of imputation models is not part of routine practice. A recent review highlighted the rapid uptake of MI in the last few years, but also identified that very few researchers check imputation models or examine the sensitivity of results to modelling decisions [11]. The failure to perform model checks may be due to the lack of guidance for performing imputation diagnostics, or the dearth of tools for performing such checks in statistical packages.

In this paper, we aim to address this gap by providing an overview of available methods for checking imputation models. In the next section, we introduce an illustrative analysis affected by missing data from the Longitudinal Study of Australian Children. We then review existing methods for checking imputation models and illustrate these techniques using the case study. We end with a discussion of the proposed model checking approaches.

## Missing data case study

The case study in this paper uses data from the Longitudinal Study of Australian Children (LSAC), a nationally representative study of childhood development [12]. LSAC is a longitudinal cohort study consisting of 5107 children recruited at 0−1 years of age (B cohort) and 4983 children recruited at 4−5 years of age (K cohort), who have been followed up every two years since 2004. Details of the study design have been described elsewhere [13].

In this paper, we use data from LSAC’s B cohort to examine the relationship between harsh parental discipline in early childhood (2–3 years) and conduct problems at 6–7 years. The outcome was assessed using the conduct subscale of the Strengths and Difficulties Questionnaire [14] with scores ranging between 0 and 10. These scores were dichotomised to produce a binary variable that was equal to 1 (i.e. “conduct problems”) if a child scored 3 or above, and 0 otherwise. The exposure of interest was measured by the hostile parenting scale [15–17], on which scores ranged between 1 and 10, with higher scores representing harsher parenting.

The following logistic regression model was used to assess the relationship between the risk of conduct problems and harsh parental discipline, with adjustment for potentially confounding parent and child factors (child sex, family socioeconomic status, financial hardship and maternal psychological distress):

$$ \text{logit}\,{p({\texttt{conduct\_bin}}) = \gamma }_{\text{0}} +{  \gamma }_{\text{1}} {{\texttt{harsh}} + \gamma }_{\text{2}} {{\texttt{sex}} + \gamma }_{\text{3}} {{\texttt{SEP}} + \gamma }_{\text{4}} {{\texttt{hardship}} + \gamma }_{\text{6}} \text{{\texttt{distress}}} $$

where conduct_bin represented conduct problems and harsh was the harsh parenting exposure variable. Sex was a binary variable where 0 = female and 1 = male, SEP was an internally standardised measure (“Z-score”) of a family’s socioeconomic position, hardship was a measure of financial stress (range 0–6) and distress was the mother’s score on the Kessler-6 scale for psychological distress (range 0–24) [18]. We refer to this logistic regression model as the “analysis model” to distinguish it from models used for imputation. Note that the example is used for illustrative purposes only, as it simplifies various aspects of the underlying substantive issues concerning parenting and child behaviour.

### Assessment of missing data

Of the 5107 children in the B cohort, 3163 (62%) children had data available for all variables in the analysis model. Eighteen percent of the study participants had missing outcome data, while 31% did not have data for the exposure of interest (harsh). The only completely observed variable was child sex. Table 1 shows the patterns of co-occurrence of missing values across the variables in the analysis. The missing data patterns do not follow a regular pattern, with many participants missing individual covariates.

**Table 1:** Missing data patterns for variables in the logistic regression analysis model (n = 5107)

Number of participants	Percent	Conduct problems	Harsh discipline	SEP	Hardship	Psychological distress
3163	62	+	+	+	+	+
733	14	+	−	+	+	+
352	7	−	−	−	−	−
255	5	−	+	+	+	+
234	5	−	−	+	+	+
149	3	+	−	−	−	−
82	2	+	−	+	+	−
55	1	+	+	+	+	−
41	1	−	−	+	+	−
22	0.4	+	+	+	−	+
7	0.1	−	+	+	+	−
5	0.1	+	−	+	−	+
3	0.1	−	−	+	−	+
2	0.04	−	+	−	+	+
1	0.02	−	−	+	−	−
1	0.02	−	+	+	−	+
1	0.02	+	−	−	+	−
1	0.02	+	+	−	+	+

Nb. + indicates value is present and − indicates value is missing. The sex variable was not included in the missing data patterns, because it was completely observed

Table 2 presents summary statistics of baseline variables for the complete and incomplete cases. Children with completely observed data differed from the incomplete cases in the following major ways: they had higher socioeconomic Z-scores on average (complete cases: mean = 0.19 vs. incomplete cases: mean = −0.31), as well as a higher percentage of mothers completing high school (74 vs. 55%) and speaking English as their primary language (90 vs. 82%). There was a smaller proportion of sole parent families among complete cases compared to incomplete cases (6 vs. 15%).

**Table 2:** Baseline characteristics of participants with complete and incomplete data for the variables in the analysis model

Variable	Complete cases (n = 3163)	Incomplete cases (n = 1944)
Mother’s age (at baseline), mean (SD)	31.8 (4.9)	29.6 (6.0)
Socioeconomic Z-score, mean (SD)	0.19 (1.0)	−0.31 (1.0)
Child sex (male), fraction (%)	1625/3163 (51.4)	983/1944 (50.6)
Indigenous status, fraction (%)	73/3163 (2.3)	157/1944 (8.1)
Mother’s main language is not English, fraction (%)	2825/3126 (90.4)	1539/1877 (82.0)
Sole parent family, fraction (%)	183/3163 (5.8)	294/1944 (15.1)
Child has a sibling, fraction (%)	1895/3163 (59.9)	1193/1944 (61.4)
Mother completed high school, fraction (%)	2350/3161 (74.3)	1060/1937 (54.7)

Nb. The denominators in the fractions are the numbers of participants for whom the measure was available

The assessment of the missing data suggests that this analysis could benefit from MI. Firstly, there is a substantial amount of missing data, with nearly all of the analysis model variables being incompletely observed. A complete case analysis would discard 38% of the sample, whereas MI can use partially-observed data from the incomplete cases. Secondly, restricting the analysis to the complete cases could produce biased results since there appear to be systematic differences between those with observed and missing data. Finally, use of MI in this analysis could take advantage of the availability of several other variables in the LSAC dataset that could be included in the imputation model.

### Proposed imputation model

After assessing the missing data and deciding that MI would be an appropriate method of analysis, the next step is to develop the imputation model that will be used to generate imputed values. Based on recommendations in the MI literature [19, 20], we included all of the variables from the analysis model in the imputation model to ensure that the imputation model preserved the relationships between the variables of interest [21, 22]. We included the continuous version of the outcome variable (conduct) in the imputation model, because it potentially contained more information than the dichotomised version (conduct_bin). After imputation, we derived the binary outcomes from the imputed values of the continuous outcome variable. We note, however, that imputing the continuous version of the outcome variable could lead to problems with the imputation model not aligning with the logistic regression analysis model, an issue to which we return later.

We also included a number of variables that were not in the analysis model (often referred to as *auxiliary variables* in the MI literature) [6]. Because LSAC is a longitudinal study, we had access to repeated measurements of the variables that were in our analysis model. These repeated measurements are good candidates for use as auxiliary variables, as they are highly correlated with our incomplete variables, and hence could be expected to improve the prediction of the missing values [6, 23]. The MI literature also recommends including predictors of missingness in the imputation model, to improve the plausibility of the MAR assumption underlying MI [3, 24]. Predictors of missingness included: mother’s age, whether the mother’s main language is English, child’s indigenous status, and whether the mother completed high school [25]. We selected 19 auxiliary variables, giving a total of 25 variables in the imputation model.

Because of the patchwork (“non-monotone”) pattern of missing values occurring in several variables, and because we needed to impute missing values for different types of variables (e.g. continuous and categorical), we decided to use multiple imputation by chained equations (MICE). In this approach missing values are imputed using a series of univariate conditional imputation models [26, 27]. We imputed continuous variables using linear regression models and binary variables using logistic regression models. Although some of the continuous variables were skewed, they were imputed on the raw scale (i.e. without transformation) irrespective of their distribution [10, 28]. MI was implemented using the *mi impute chained* command in Stata software version 14.1 [29]. We generated 40 imputed (“completed”) datasets based on the rule of thumb that the number of imputations should be at least equal to the percentage of incomplete cases (which was 38% in this case) [19].

## Checking the imputation model

In this section, we provide an overview of currently available methods for checking imputation models, ranging from simple graphical displays of the data through to complex simulation-based methods. These model checking approaches are illustrated using the LSAC case study.

### Exploring the imputed values

A useful initial check is to explore the imputed values that have been generated by the imputation model. This can be done using graphical displays of the imputed data using plots such as histograms or boxplots. The imputed data can also be checked numerically by generating descriptive statistics. These graphical and numerical checks provide information about the distribution of imputed values, and can be useful for assessing whether the imputed data are reasonable.

Judgements about the plausibility of the imputed data should be made with respect to subject matter knowledge. Abayomi et al. [30] characterise such diagnostics as *external* checks, since the model is being evaluated with respect to information external to the data at hand. Imputed data that are extremely implausible given subject matter knowledge could signal a potential problem with the imputation model.

However, it is also important to keep in mind that the goal of MI is not to recover or replace the missing values, rather it is to produce valid analytic results in the presence of missing data. Simulation studies have indicated that it is not essential that imputed values fall within plausible or possible ranges [28, 31]. For example, considering missing values in our harsh discipline variable, it may not be problematic if imputed values fall outside the range of possible scores on the harsh parenting scale. Given that our interest lies in associations between harsh parenting and child behaviour, it may be more important that relationships between variables are preserved during the imputation process.

### Comparisons between observed and imputed data

**Fig. 1:**
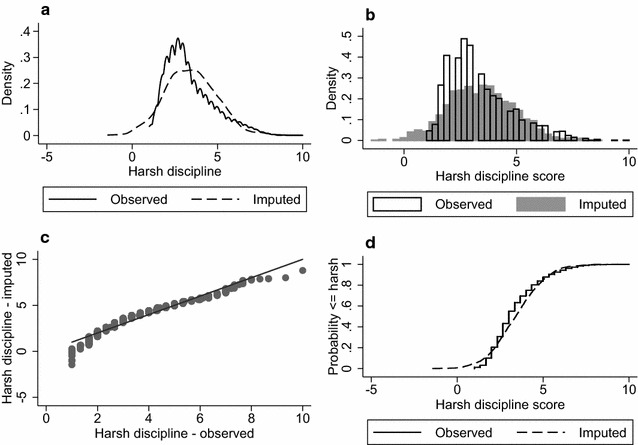
Graphs comparing the distributions of the observed (n = 3506) and imputed (n = 1601) harsh discipline scores. **a** Kernel density plot of the observed (*solid line*) and imputed (*dashed line*) harsh discipline scores, **b** histogram of the observed (*transparent bars*) and imputed (*grey bars*) harsh discipline scores, **c** plot of the quantiles of the imputed harsh discipline scores against quantiles of the observed scores (quantile–quantile plot), and **d** cumulative distribution plots of the observed (*solid line*) and the imputed (*dashed line*) harsh discipline scores. Data from a single imputed dataset have been presented

**Fig. 2:**
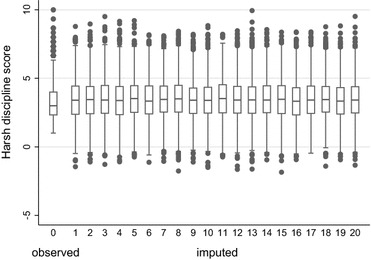
Boxplots of the observed (labelled 0) and imputed harsh discipline scores (labelled 1–20). Data are shown for the first 20 imputed datasets

One of the commonly recommended diagnostics is a graphical comparison of the observed and imputed data [19, 20, 30, 32]. These comparisons can be considered an *internal check*, as the data are being assessed with respect to available data [30]. Recommended plots for comparing observed and imputed data include histograms [33], boxplots [19], density plots [30], cumulative distribution plots [34], strip plots [20] and quantile–quantile plots [32]. Figure 1 presents four plot types for comparing observed and imputed harsh parental discipline scores for a single imputed dataset. Figure 1a (kernel density plot) and b (histogram) demonstrate that the observed data are positively skewed, while the distribution of the imputed values is symmetrical. The quantile–quantile and cumulative distribution plots in Fig. 1c, d shows alternative comparisons of the distribution of observed and imputed values which do not readily highlight this difference in the distributions. Figure 2 shows a boxplot of the observed data (labelled 0) alongside the imputed data for the first 20 imputations (labelled 1–20). This type of plot enables each of the imputed datasets to be viewed separately in a single figure. Again, the boxplots reveal some differences between the observed and imputed data, including a more symmetrical distribution for the imputed data, and slightly higher median values in the imputed data compared to the observed data.

When working with multiple incomplete variables, it is not always feasible to perform graphical checks of all imputed variables and all sets of imputations. An alternative approach is to tabulate summary statistics of the observed and imputed data (Table 3). In our case study, the observed and imputed harsh discipline scores had similar means (3.36 vs. 3.44) and standard deviations (1.44 vs. 1.47). However, there were discrepancies between the observed and imputed values of other variables, including differences in mean socioeconomic position (observed = 0.0, imputed = −0.51), and mean psychological distress score (observed = 2.93, imputed = 3.70).

**Table 3:** Summary statistics of the observed and imputed data for the incomplete variables in the analysis model

	Observed					Imputed				
	N	Mean	SD	Min	Max	N	Mean	SD	Min	Max
Harsh discipline	3506	3.36	1.44	1	10	1601	3.44	1.47	−2.73	9.94
Socioeconomic position	4602	0.00	1.00	−4.90	3.03	505	−0.51	1.03	−5.24	3.20
Financial hardship	4574	0.29	0.71	0	6	533	0.46	0.77	−2.36	3.94
Psychological distress	4419	2.93	3.24	0	24	688	3.70	3.49	−9.01	19.87
Conduct problems	4211	21.5%^a^				896	20.1%^a^			

The summary statistics of the imputed data were calculated using pooled data over 40 imputations

*SD* standard deviation, *Min* minimum, *Max* maximum

^a^Percent with characteristic

Some authors have proposed using formal numerical methods to compare the distributions of observed and imputed values, in order to highlight variables that may be of concern. For example, Stuart et al. [32] proposed comparing the means and variances of observed and imputed values. They suggested flagging variables if the ratio of variances of the observed and imputed values is less than 0.5 or greater than 2, or if the absolute difference in means is greater than two standard deviations. Abayomi et al. [30] proposed using the Kolmogorov–Smirnov test to compare the empirical distributions of the observed and imputed data, and they flagged variables as potentially concerning if they had a p value below 0.05. Although such numerical tests provide an expedient means for checking a large number of imputed variables, the results can be difficult to interpret, because the magnitude of the p-values depends on both the sample size and the proportion of missing values in the incomplete variables [35].

It is also important to recognise that discrepancies between observed and imputed data are not necessarily problematic, since under MAR we may expect such differences to arise. To interpret whether these discrepancies could be problematic, one can draw on external information [30, 32, 33]. Imputers should consider whether observed discrepancies are to be expected given what is known about the incomplete variables and the missing data process. For example, in the case of LSAC it is known that lower socioeconomic position is associated with missingness, so we would expect the imputed socioeconomic scores to be lower than the observed [36].

**Fig. 3:**
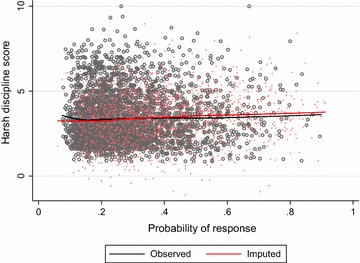
Scatterplot of the harsh discipline scores against the estimated probabilities of response with lowess curves. Data shown are observed values (*black*) and imputed values (*red*) for one imputed dataset only

Bondarenko and Raghunathan [37] suggested comparing the observed and imputed distributions conditional on the propensity of response for that variable. Under MAR mechanisms, this is a potentially more useful strategy, as we expect the observed and imputed data to be similar conditional on the response probability. To check the imputed values of harsh discipline, we estimated probabilities of response using a logistic regression model with the missing data indicator as the outcome variable and completed variables as predictors (this was done separately for each imputed dataset). We then checked the imputations graphically by plotting the harsh discipline scores against the estimated response propensity, using different coloured markers for the observed and imputed data (Fig. 3). The plot can be examined for major differences in the distribution of observed and imputed data for a given value of the propensity score [37]. Figure 3 suggests slight differences in the shapes of the distributions of the observed and imputed harsh discipline values (conditional on response propensity), with the observed distribution being less symmetrical. However, the means of the observed and imputed values are conditionally very similar.

It is also possible to perform more formal checks after grouping individuals into strata according to their estimated probabilities of response. For example, Bondarenko and Raghunathan [37] propose checking continuous variables using analysis of variance (ANOVA) where the outcome variable is the variable being imputed and the factors are the response stratum, the indicator for observed/imputed status and their interaction. Based on empirical results from simulations, Bondarenko and Raghunathan [37] suggest rejecting an imputation model if the ANOVA test is rejected in 2 of 5 imputed datasets (using an alpha level of 0.05). We performed an ANOVA on each of the 40 imputed datasets; in 7 of the 40 imputed datasets the p-value for the interaction was <0.05, and in 2 of the 40 datasets the p-value for main effect for missingness indicator was <0.05. Based on these checks, the imputation model appeared to be adequate for the imputation of the harsh discipline variable.

### Standard regression diagnostics

**Fig. 4:**
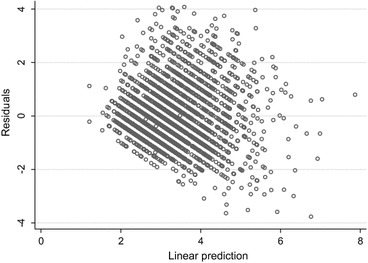
Plot of the residuals against the predicted values for the proposed imputation model fitted to the observed data for harsh discipline

Imputation models are often based on regression models, either when imputing a single incomplete variable, or within a sequence of univariate regression imputation models using MICE as described above [26]. In this context, it is natural to check the goodness-of-fit of the imputation models using established methods for checking assumptions of regression models. Standard regression diagnostics include investigations of residuals, outliers and influential cases. Marchenko and Eddings [38] suggest fitting the proposed regression imputation model to the observed data prior to performing MI, and then performing regression diagnostics. If the diagnostics suggest poor model fit, then the imputation model could be modified before generating the imputations. For example, Fig. 4 shows a plot of residuals against fitted values for the linear regression imputation model for harsh discipline score (applied to the observed data, i.e. prior to imputation). This plot can be used to check the assumptions of the linearity of the regression function and the homogeneity of error variance. There is some striation in the plot (due to many of the harsh discipline scores taking on integer values), but on the whole, the residuals appear to have constant variance and do not display any trends across the range of fitted values.

If the substantive analysis is a regression analysis, then it is also possible to perform standard regression diagnostics for the *analysis model* after imputation. These diagnostics are primarily a check of the fit of the analysis model, but they can be used to check for differences in the model fit across the multiple completed datasets. After performing MI, residuals can be generated for each completed dataset. For individuals with observed data, the residual is calculated as the difference between the observed value and its prediction from the analysis model; while for those with imputed data, the residual is the difference between the imputed value and its prediction from the analysis model. The residuals can then be plotted against the fitted values for each completed dataset. White et al. [19] suggested that, if problems (e.g. outliers) occurred in only a few of the residual plots, then this might indicate a problem with the imputation model. If, however, the extreme values were consistent across all datasets, then the problems could be attributed to the analysis model.

### Cross-validation

Checking of imputation models can also be performed by cross-validation, which assesses the predictive ability of a model. In *leave*-*one*-*out cross*-*validation*, a single observation is deleted and the proposed model is fitted to the remaining data and used to predict the outcome for the excluded data point. This process is repeated by cycling through each observation, deleting and predicting the outcome for each observation in turn. The predictive performance of the model can be assessed numerically by summarising the discrepancies between the observed and predicted outcome values. The model can also be assessed graphically by plotting the predicted values against the observed values [39].

**Fig. 5:**
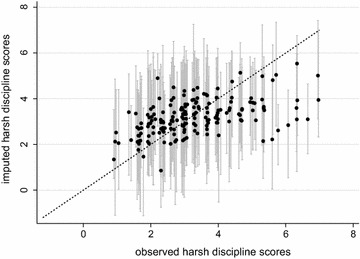
Leave-one-out cross-validation plot for the harsh discipline scores. The median imputed values across 20 imputations (*black markers*) have been plotted against the observed value. The *error bars* are the intervals between the 5th and 95th percentiles

Figure 5 shows a leave-one-out cross-validation plot for the harsh discipline score. To produce this graph, the observed values of harsh were deleted in turn and imputed using 20 imputations. Because there were 3506 participants with observed harsh discipline values, the plot could have been generated based on 3506 cycles of deletion and imputation. To reduce the computational burden, this cross-validation plot was produced using a random selection of 10% of the observations. In Fig. 5, the median imputed values (calculated over 20 imputed datasets) have been plotted against the observed values. The error bars span between the 5th and 95th percentiles of the imputed values. The markers were jittered in the x-direction, because many participants shared the same observed harsh discipline values.

In Fig. 5, the prediction intervals do not always contain the observed values. At the lower end of the harsh discipline scale, the imputation model overestimates the scores, while at higher values of harsh discipline, the scores tend to be underestimated. This suggests that the imputation model has poorer predictive performance at the extreme values.

### Posterior predictive checking

One final method that has been proposed for checking an imputation model is posterior predictive checking (PPC) [40–43]. This is a Bayesian model checking technique that involves simulating “replicated” datasets from the proposed imputation model [40] (see He and Zaslavsky [42] for a practical method for generating replicated datasets using standard MI routines).

An important feature of PPC is that it is designed to investigate the potential effect of model inadequacies on the ultimate results of interest (rather than focussing on the intermediate step of the quality of the imputed data values). This is done by comparing inference from the completed data (consisting of observed and imputed data) to the inference from the replicated data (drawn entirely from the imputation model). The premise of PPC is that if the model were a good fit to the data, then analyses of the completed and replicated datasets should yield similar results.

To assess an imputation model using PPC, one or more *test quantities* are selected; these test quantities are generally parameters of scientific interest. For example, if the analysis model were a regression model, the test quantities could be regression coefficients, standard errors and p-values. To test model fit using PPC, after simulating replicated datasets from the imputation model, the test quantities are estimated in both the replicated and completed datasets. Systematic differences in the estimates from the completed data and replicates may indicate poor model fit with respect to the chosen test quantities.

The discrepancy between the completed and replicated data can be summarised using the so-called *posterior predictive p*-*value*, which is defined as the probability that the replicated data are more extreme than the completed data with respect to the chosen test quantity [42]. The posterior predictive p-values can be estimated as the proportion of replications in which the estimate of the test quantity from the replicated data is larger than that estimated from the completed data. Posterior predictive p-values that are close to 0 or 1 indicate systematic differences, and potential problems with the imputation model.

For the LSAC example, we performed PPC using as test quantities the estimated coefficients from the logistic regression analysis. We created 2000 replications and calculated means of the test quantities in the replicated and completed data. The discrepancies between the completed and replicated data were then summarised both graphically and numerically. In Table 4 we present summary statistics of the estimates of the test quantities in the completed and replicated data. $\bar{T}(Y_{com} )$ and $\bar{T}(Y_{com}^{rep} )$ represent the (posterior predictive) means of the test quantities across 2000 completed and replicated datasets, respectively. For example, for the regression coefficient for harsh discipline, the estimated mean in the completed datasets was $\bar{T}(Y_{com} ) = 0.31$ (corresponding to an odds ratio = 1.36), while the estimated mean in the replicated datasets was $\bar{T}(Y_{com}^{rep} ) = 0.26$ (odds ratio = 1.30).

**Table 4:** Results of posterior predictive checking for the logistic regression coefficients

Test quantity (regression coefficient)	Initial imputation model^a^			Updated imputation model^b^		
	$\bar{T}(Y_{com} )$	$\bar{T}(Y_{com}^{rep} )$	PPP	$\bar{T}(Y_{com} )$	$\bar{T}(Y_{com}^{rep} )$	PPP
Harsh discipline	0.31	0.26	0.026	0.31	0.33	0.71
Sex	0.39	0.38	0.45	0.39	0.37	0.44
Socioeconomic position	−0.31	−0.3	0.63	−0.34	−0.34	0.53
Financial hardship	0.08	0.1	0.63	0.09	0.11	0.63
Psychological distress	0.04	0.06	0.94	0.04	0.05	0.62

Posterior predictive p values (PPP) are shown along with means of the test quantities (regression coefficients) estimated in the completed datasets, $\bar{T}(Y_{com} )$, and the replicated datasets, $\bar{T}(Y_{com}^{rep} )$. Results are based on 2000 replications

^a^The initial imputation model included the outcome variable as a continuous variable

^b^The updated imputation model included the binary version of the outcome variable that was also used in the analysis

**Fig. 6:**
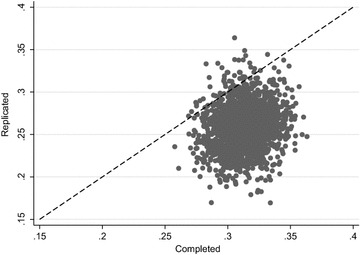
Posterior predictive checks of the coefficient for harsh discipline from the logistic regression analysis model. Estimates of the regression coefficient for harsh discipline from the replicated data are plotted against the estimates from the completed data (based on 2000 replications). The proportion of markers above the y = x line represents the posterior predictive p value (PPP = 0.026)

Table 4 also displays the posterior predictive p-value for each of the test quantities. The PPC results point to potential model inadequacies with respect to the logistic regression analysis. The posterior predictive p-value for the regression coefficient for harsh discipline was 0.026; thus, in 2.6% of the 2000 replications, the estimate in the replicated dataset was larger than that obtained from the actual data, suggesting poor model fit. These results are also displayed visually in Fig. 6, which is a scatterplot of the estimated regression coefficient for harsh discipline in the replicated data plotted against the estimated regression coefficient in the completed data. The proportion of points above the $y = x$ line corresponds to the posterior predictive p-value (i.e. 0.026).

The graphical and numerical PPC results revealed a potential lack of fit of the imputation model with respect to the logistic regression analysis. These PPC results drew our attention to a problem with the proposed imputation model; the imputation model was incompatible with the logistic regression analysis, in the sense that the continuous version of the outcome variable (conduct) was included in the imputation model rather than the binary outcome that was used in the analysis (conduct_bin). The imputation model fitted linear relationships between the conduct outcome variable and the covariates, whereas a threshold relationship was the analysis of interest. As a result of these checks, we repeated the imputation, using conduct_bin instead of conduct in the imputation model, and found that the PPC p-values became less extreme, i.e. moved closer to 0.5 (see Table 4).

### Availability of model checking tools

To date very few imputation diagnostics have been made available in statistical software. At the time of writing, add-on packages for R offered the widest range of imputation diagnostics. For example, the mi, mice and Amelia packages include features for model checking in addition to their core functions for imputing missing values [33, 44, 45]. The VIM and miP packages in R have been designed specifically for visualising imputed data [46, 47]. All of these packages have functions for graphically comparing the distributions of the observed and imputed data. Some of these packages also offer scatterplots for plotting the observed and imputed data against another variable [33, 44, 46, 47]. The mi package has tools for producing residual plots for checking imputation models when imputing data using MICE [33]. The Amelia software also has a diagnostic feature called “overimputation”, which generates cross-validation plots of the mean imputed values against the observed values with 90% confidence intervals [45].

There are very few imputation diagnostics available in the popular commercial packages. For example, at the time of writing this paper, SAS [48] and Stata [29] did not have built-in features for performing imputation diagnostics (besides checks of convergence). However, in Stata there is a user-written command, midiagplots, for producing graphical diagnostics [34]. This command has features for comparing the imputed and the observed data using plots such as kernel density plots. Although diagnostic features have not yet been incorporated into many statistical packages, it is possible to write syntax to perform many of these checks (see Additional file 1).

## Conclusions

In this paper, we have provided an overview of a number of proposed diagnostics for checking of imputation models, from simple descriptive methods through to more complex approaches such as cross-validation and posterior predictive checking. A summary of the model checking approaches is shown in Table 5.

**Table 5:** Overview of approaches to model checking in multiple imputation

*Consider the plausibility of the imputed data*
Explore imputed values using descriptive statistics and graphical displays
Use subject matter knowledge to judge the plausibility of imputed values, but remember that imputed values do not necessarily have to resemble observed data, as the goal of MI is not to predict the missing values but to produce valid inference in the presence of missing data
*Comparisons of observed and imputed data*
The imputed data should be compared with the observed data to assess plausibility and identify major problems with the imputation model
Comparisons can be made using summary statistics and graphical methods
Discrepancies between observed and imputed data do not necessarily signal a problem under MAR, but should be judged for their plausibility under likely missingness processes
*Consider the analysis of interest*
Consider the target analysis when making judgements about model adequacy. If one is interested in characteristics of the marginal distributions (e.g. percentiles), then it might be important that features of the marginal distributions are preserved in the imputed data. This becomes less critical if the primary interest lies in relationships between variables
Posterior predictive checking can be used to check the adequacy of imputation models with respect to quantities of substantive interest. Model fit can be explored using either graphical or numerical summaries (e.g. posterior predictive p-values), but again there can be no hard and fast rules for determining adequacy of model specification
*Take a multifaceted approach*
Use a number of different diagnostics to check imputation models. For example, descriptive statistics can be used to check the quality of imputed values themselves, while methods such as posterior predictive checking can be used to assess the imputation model with respect to target analyses

We illustrated the model checking techniques using a case study of parenting and child behaviour. The model checks in the case study drew our attention to potential problems with our imputation model. In particular, the PPC diagnostic flagged an important issue regarding the omission of the binary outcome variable from the imputation model. This was a reminder of the importance of compatibility between the imputation and analysis models, and the need to tailor imputation models for the analysis at hand [49].

Although we illustrated a number of diagnostic methods, they all have strengths and weaknesses. The graphical checks were useful for exploring the imputed values, but it can be challenging to apply them routinely to all imputed variables when working with large numbers of incomplete variables. Comparisons of the observed and imputed data can be used to identify discrepancies between the observed and imputed data, but these comparisons can be difficult to interpret when data are suspected to be MAR. PPC is preferable to methods that focus on the plausibility of imputations, because it checks models with respect to quantities of substantive interest. In general, we suggest treating each of the techniques presented in this paper as separate elements of a diagnostic toolkit.

In this paper, we assumed that the missing data mechanism is MAR and that an MI analysis under MAR would be less biased than a complete case analysis. However, we acknowledge that there are scenarios under which MI can also produce biased results even when data are MAR (as illustrated in [50]). Unfortunately it difficult to identify such scenarios in practice when working with complex multivariate missing data problems. In addition, it is not possible to check the validity of the MAR assumption without knowing the values of the missing data. Thus, in addition to performing diagnostic checks, it is also important to examine whether results change under different assumptions concerning the missing data mechanisms. This is an ongoing area of research, with pattern mixture methods [51] and weighting approaches [52] being proposed methods of analysis when data are suspected to be not missing at random.

Given the increasing popularity of MI and the availability of automated tools for generating imputations, we echo the concerns of others that greater attention should be paid to methods for checking imputation models [30, 42]. Our overview of currently proposed methods for model checking highlights the need for further research on this topic, in particular to develop better understanding of how useful each of these methods is for detecting problems with imputation models. Such work should encourage the development of both computational tools and guidance for carrying out imputation model checks, which are needed to promote the sensible implementation of MI. This will become increasingly important as MI becomes further established as a standard missing data method into the future.

## Additional file

**Additional file 1.** Example Stata code for performing checks of multiple imputation models

## Abbreviations

ANOVA: analysis of variance
LSAC: Longitudinal Study of Australian Children
MAR: missing at random
MI: multiple imputation
MICE: multiple imputation by chained equations
PPC: posterior predictive checking

## References

[1] Little RJ, D’Agostino R, Cohen ML, Dickersin K, Emerson SS, Farrar JT, Frangakis C, Hogan JW, Molenberghs G, Murphy SA (2012). The prevention and treatment of missing data in clinical trials. N Engl J Med.

[2] Rubin DB (1987). Multiple imputation for nonresponse in surveys.

[3] Sterne JAC, White IR, Carlin JB, Spratt M, Royston P, Kenward MG, Wood AM, Carpenter JR (2009). Multiple imputation for missing data in epidemiological and clinical research: potential and pitfalls. BMJ.

[4] Kenward MG, Carpenter J (2007). Multiple imputation: current perspectives. Stat Methods Med Res.

[5] Lee KJ, Carlin JB (2010). Multiple imputation for missing data: fully conditional specification versus multivariate normal imputation. Am J Epidemiol.

[6] Collins LM, Schafer JL, Kam CM (2001). A comparison of inclusive and restrictive strategies in modern missing data procedures. Psychol Methods.

[7] Seaman S, Bartlett J, White I (2012). Multiple imputation of missing covariates with non-linear effects and interactions: an evaluation of statistical methods. BMC Med Res Methodol.

[8] Lee KJ, Galati JC, Simpson JA, Carlin JB (2012). Comparison of methods for imputing ordinal data using multivariate normal imputation: a case study of non-linear effects in a large cohort study. Stat Med.

[9] Yucel RM, He Y, Zaslavsky AM (2011). Gaussian-based routines to impute categorical variables in health surveys. Stat Med.

[10] Lee KJ, Carlin JB (2017). Multiple imputation in the presence of non-normal data. Stat Med.

[11] Hayati Rezvan P, Lee KJ, Simpson JA (2015). The rise of multiple imputation: a review of the reporting and implementation of the method in medical research. BMC Med Res Methodol.

[12] Australian Institute of Family Studies. Longitudinal Study of Australian Children Data User Guide. Melbourne; 2011.

[13] Nicholson J, Sanson A, Ungerer J, Wilson K, Zubrick S. Introducing the Longitudinal Study of Australian Children—LSAC discussion paper no. 1. Edited by Australian Institute of Family Studies; 2002.

[14] Goodman R (1997). The Strengths and Difficulties Questionnaire: a research note. J Child Psychol Psychiatry.

[15] National Center for Education Statistics (2004). Early Childhood Longitudinal Study (ECLS).

[16] Statistics Canada (2000). National Longitudinal Survey of Children and Youth (NLSCY) Cycle 3 survey instruments: parent questionnaire.

[17] Zubrick SR, Lucas N, Westrupp EM, Nicholson JM. Parenting measures in the Longitudinal Study of Australian Children: Construct validity and measurement quality, waves 1 to 4. Canberra; 2014.

[18] Kessler RC, Barker PR, Colpe LJ (2003). Screening for serious mental illness in the general population. Arch Gen Psychiatry.

[19] White IR, Royston P, Wood AM (2011). Multiple imputation using chained equations: issues and guidance for practice. Stat Med.

[20] van Buuren S (2012). Flexible imputation of missing data.

[21] Schafer JL (1997). Analysis of incomplete multivariate data.

[22] Moons KGM, Donders RART, Stijnen T, Harrell FE (2006). Using the outcome for imputation of missing predictor values was preferred. J Clin Epidemiol.

[23] Graham JW (2012). Missing data: analysis and design.

[24] Schafer JL, Olsen MK (1998). Multiple imputation for multivariate missing-data problems: a data analyst’s perspective. Multivar Behav Res.

[25] Soloff C, Lawrence D, Misson S, Johnstone R. LSAC technical paper no. 3: Wave 1 weighting and non-response; 2006.

[26] van Buuren S (2007). Multiple imputation of discrete and continuous data by fully conditional specification. Stat Methods Med Res.

[27] Raghunathan TE, Lepkowski JM, Van Hoewyk J, Solenberger P (2001). A multivariate technique for multiply imputing missing values using a sequence of regression models. Surv Methodol.

[28] von Hippel PT (2013). Should a normal imputation model be modified to impute skewed variables?. Sociol Methods Res.

[29] StataCorp. Stata statistical software: release 14. College Station: StataCorp LP; 2015.

[30] Abayomi K, Gelman A, Levy M (2008). Diagnostics for multivariate imputations. J R Stat Soc Ser C Appl Stat.

[31] Rodwell L, Lee K, Romaniuk H, Carlin J (2014). Comparison of methods for imputing limited-range variables: a simulation study. BMC Med Res Methodol.

[32] Stuart EA, Azur M, Frangakis C, Leaf P (2009). Multiple Imputation with large data sets: a case study of the children’s mental health initiative. Am J Epidemiol.

[33] Su YS, Gelman A, Hill J, Yajima M (2011). Multiple imputation with diagnostics (mi) in R: opening windows into the black box. J Stat Softw.

[34] Eddings W, Marchenko Y (2012). Diagnostics for multiple imputation in Stata. Stata J.

[35] Nguyen CD, Carlin JB, Lee KJ (2013). Diagnosing problems with imputation models using the Kolmogorov–Smirnov test: a simulation study. BMC Med Res Methodol.

[36] Sipthorp M, Misson S. LSAC technical paper no. 6: Wave 3 weighting and non-response; 2009.

[37] Bondarenko I, Raghunathan T (2016). Graphical and numerical diagnostic tools to assess suitability of multiple imputations and imputation models. Stat Med.

[38] Marchenko YV, Eddings W. A note on how to perform multiple-imputation diagnostics in Stata. 2011. http://www.stata.com/users/ymarchenko/midiagnote.pdf.

[39] Gelman A, King G, Liu CH (1998). Not asked and not answered: multiple imputation for multiple surveys. J Am Stat Assoc.

[40] Gelman A, Carlin JB, Stern HS, Dunson DB, Vehtari A, Rubin DB (2013). Bayesian data analysis.

[41] Nguyen CD, Lee KJ, Carlin JB (2015). Posterior predictive checking of multiple imputation models. Biom J.

[42] He Y, Zaslavsky AM (2011). Diagnosing imputation models by applying target analyses to posterior replicates of completed data. Stat Med.

[43] Gelman A, Van Mechelen I, Verbeke G, Heitjan DF, Meulders M (2005). Multiple imputation for model checking: completed-data plots with missing and latent data. Biometrics.

[44] Van Buuren S, Groothuis-Oudshoorn K (2011). mice: multivariate imputation by chained equations in R. J Stat Softw.

[45] Honaker J, King G, Blackwell M (2011). Amelia II: a program for missing data. J Stat Softw.

[46] Templ M, Alfons A, Kowarik A, Prantner B. VIM: visualization and imputation of missing values. Version 4.0 ed; 2013.

[47] Brix P. miP: multiple imputation plots. Version 1.1 ed; 2012.

[48] SAS Institute Inc. SAS/STAT^®^ 13.1 User’s Guide. Cary: SAS Institute Inc; 2013.

[49] Bartlett JW, Seaman SR, White IR, Carpenter JR, for the Alzheimer’s Disease Neuroimaging Initiative (2013). Multiple imputation of covariates by fully conditional specification: Accommodating the substantive model. Stat Methods Med Res.

[50] White IR, Carlin JB (2010). Bias and efficiency of multiple imputation compared with complete-case analysis for missing covariate values. Stat Med.

[51] Ratitch B, O’Kelly M, Tosiello R (2013). Missing data in clinical trials: from clinical assumptions to statistical analysis using pattern mixture models. Pharm Stat.

[52] Hayati Rezvan P, White IR, Lee KJ, Carlin JB, Simpson JA (2015). Evaluation of a weighting approach for performing sensitivity analysis after multiple imputation. BMC Med Res Methodol.

